# Risk of multiple sclerosis in patients with psoriasis receiving anti‐IL‐17 agents: A case‐based review

**DOI:** 10.1111/1346-8138.17143

**Published:** 2024-02-12

**Authors:** Sotirios G. Tsiogkas, Vaia Tsimourtou, Kleoniki Chaidaki, Efthymios Dardiotis, Angeliki Victoria Roussaki‐Schulze, Dimitrios P. Bogdanos, Efterpi Zafiriou

**Affiliations:** ^1^ Department of Rheumatology and Clinical Immunology, Faculty of Medicine University of Thessaly Larissa Greece; ^2^ Department of Neurology, Faculty of Medicine University of Thessaly Larissa Greece; ^3^ Department of Dermatology, Faculty of Medicine University of Thessaly Larissa Greece

**Keywords:** anti‐IL‐17, demyelination, MS, psoriasis

## Abstract

Biologics approved for psoriasis exhibit favorable safety profiles, and serious adverse events have rarely been reported. In this report, we present the case of a patient treated with ixekizumab, an anti‐interleukin (IL)‐17 agent, who 8 months later developed multiple sclerosis (MS). We also review the available literature regarding the use of anti‐IL‐17 agents in the context of psoriasis and pre‐existing or new‐onset demyelination. Eight case reports were evaluated as relevant and are presented in our report. In most of the cases secukinumab or ixekizumab administration adequately controlled both skin and pre‐existing neurological clinical manifestations. However, there has been a report of MS exacerbation under secukinumab treatment and the occurrence of myelitis in a patient receiving ixekizumab. While the anti‐IL‐17‐biologic‐mediated induction of inflammatory events in the central nervous system has not been proven and a causal relationship is lacking, such a probability should be considered in extremely rare cases.

## 1 INTRODUCTION

Antibodies that inhibit the tumor necrosis factor (TNF), the interleukin (IL) ‐17, the IL‐23 and the IL‐12/IL‐23 axes have been proven effective in inducing primary endpoints in randomized controlled trials on psoriasis.

While the use of biologics has shown favorable safety profiles, serious adverse events have also been reported. Over the last decade, TNF inhibitors (TNFi) have been suggested to induce the development of inflammatory central nervous system (CNS) events in increased ratios.^1^ Demyelinating TNFi‐induced events have also been recorded in the peripheral nervous system,^2^ however, no clear causal relationship has been established.

A few cases of serious neurological events have been published for patients treated with other biologics. For instance, ustekinumab, a monoclonal antibody blocking the p40 subunit of IL‐12 and IL‐23, has been suggested to induce posterior reversible leukoencephalopathy^3^ and demyelination.^4^ Furthermore, another report has described a case of axonal sensorimotor polyneuropathy after the initiation of guselkumab (an anti‐IL23 monoclonal antibody) for the treatment of psoriasis.^5^

In this report we present the case of a patient with psoriasis treated with ixekizumab who developed multiple sclerosis (MS). We also review the available literature regarding the use of anti‐IL‐17 agents in the context of psoriasis and pre‐existing or new‐onset demyelination. As we will discuss, puzzling in our case as a co‐precipitating factor is the fact that three months before the development of MS our patient had received a second dose of the mRNA vaccine against SARS‐CoV‐2.

## 2 CASE REPORT

A 31‐year‐old Caucasian male with a 5‐year history of psoriasis vulgaris with lesions on the face, scalp and the trunk, and a 2‐year history of nail psoriasis was referred to our department in March 2021. The patient had previously received various topical agents. He had been a smoker for the past 18 years and had been consuming moderate amounts of alcohol. The patient reported a family history of psoriasis. A psoriasis area severity index (PASI) of 15, a body surface area (BSA) of 15%, a nail psoriasis severity index (NAPSI) of 35, and a dermatology life quality index (DLQI) of 12 were measured. It was decided to initiate on‐label ixekizumab treatment for the patient.

Significant improvement on the lesions was observed 2 months later and a decrease of the PASI to 2 was measured. In July 2021, the good clinical response continued and a 100% improvement of the PASI was achieved. Moreover, a DLQI of 0 was noted. In August 2021, the patient received a second dose of the Pfizer‐BioNTech mRNA vaccine against SARS‐CoV‐2. A PASI of 0 was maintained for the next months. In October 2021, the patient experienced finger joint pain and cervical spine (facet) joint pain during the evening hours for a few days, specifically in the 2nd and 3rd finger in the metacarpophalangeal joints (distal interphalangeal, proximal interphalangeal) of the right hand (he is right‐handed), without swelling or enthesitis, which were attributed to physical exercise by an orthopedist. The symptoms subsided with non‐steroidal anti‐inflammatory drugs (naproxen 500 mg twice daily) within 2 days. Τhere were no symptoms or clinical signs that would suggest psoriatic arthritis and the patient had reported no clinical symptoms compatible with psoriatic arthritis in the past.

**FIGURE 1:**
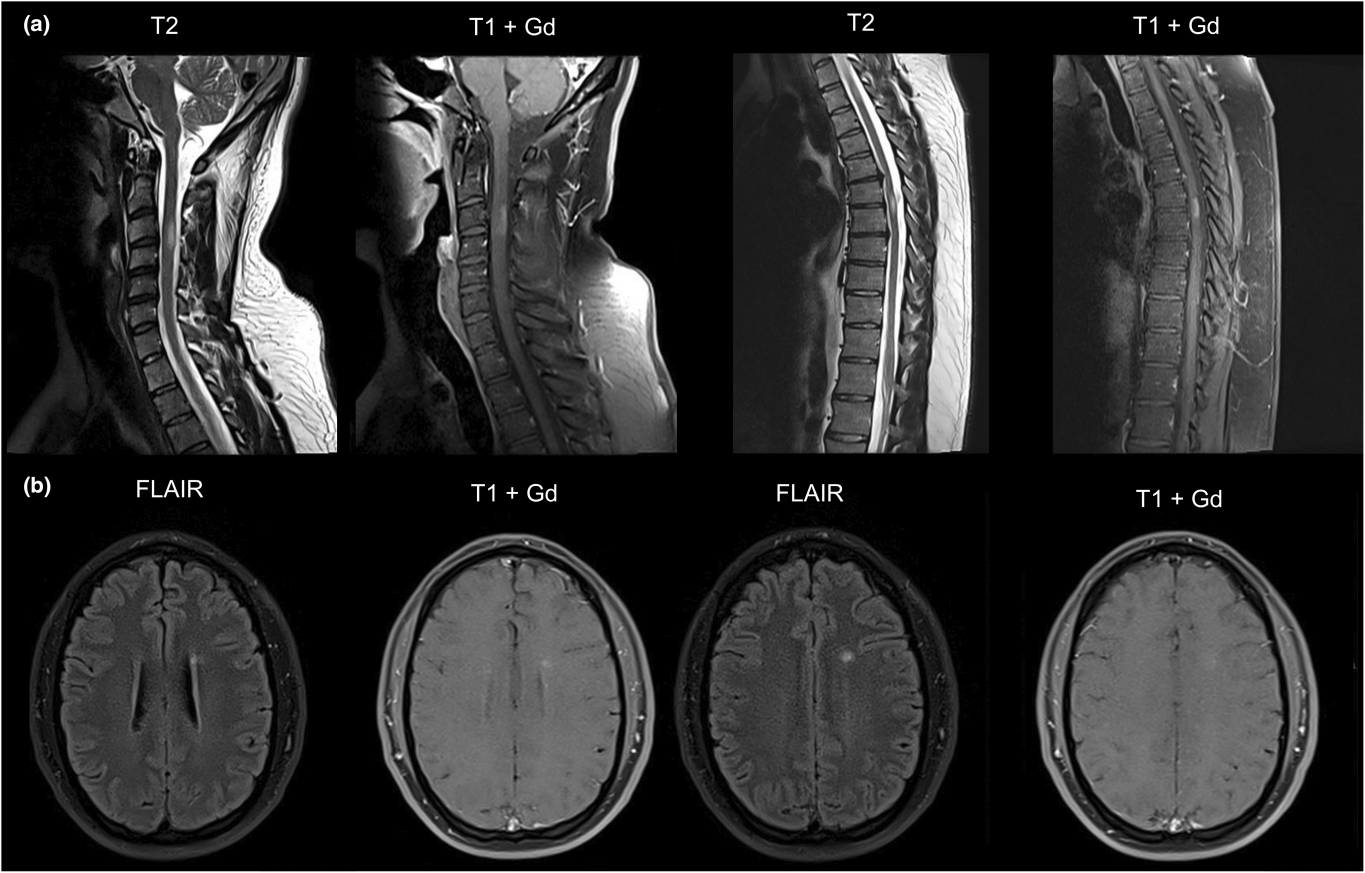
Magnetic resonance imaging of the spinal cord and the brain of the patient. (a) Hyperintense lesions are detected on both the cervical and the thoracic spine. (b) Periventricular lesion detected on the brain scans.

One month later, in November 2021 he experienced numbness of the left upper and both lower extremities. The patient was admitted to the Neurology Department for consultation. Neurological examination revealed mild congenital nystagmus and brisk tendon reflexes on the lower limbs. There was no family history of demyelinating diseases. Magnetic resonance imaging (MRI) demonstrated one hyperintense T2 lesion on the white matter of the left frontal lobe and four hyperintense T2 lesions on the cervical and thoracic spinal cord, some of which were enhanced by gadolinium (Gd‐DTPA), as shown in Figure 1. Oligoclonal bands were detected in the cerebrospinal fluid. These findings suggested a new onset demyelinating disease which was diagnosed as relapsing remitting MS (RRMS), according to the revised McDonald criteria of 2017.^6^

Ixekizumab was discontinued. The patient was initially treated with intravenous steroids and clinical improvement was achieved. Dimethyl fumarate (DMF) was initiated as treatment for both psoriasis and MS. Gradual improvement of the clinical manifestations was observed. On last follow up, 1 year after DMF initiation, there were no relapses and no further radiological deterioration.

## 3 DISCUSSION

### 3.1 Literature review

We conducted a narrative literature review. We searched the available literature through PubMed from inception until September 2023. We used relevant terms, such as “anti‐IL‐17”, “demyelinating”, “multiple sclerosis”, “optic neuritis”, and “myelitis”. In total, eight case reports corresponding to eight patients were evaluated as relevant (Table 1).

**TABLE 1:** Cases reported on anti‐IL‐17 agents in the context of psoriasis and pre‐existing or new‐onset demyelination.

First author (year)	Sex	Age	Disease	Psoriasis duration,* years	Previous treatments	Agent	Indexed neurologic condition	New onset of indexed neurologic condition while under anti‐IL‐17 treatment?	Combined therapy	Outcomes^a^ (timepoint after treatment initiation)	Comment
Assefa et al. (2019)^7^	M	47	Ps, PsA	23	Tacrolimus, UST	SEC	MS	No	Prednisolone, MTX	PASI100; joint pain reduced, stable MS [follow‐up 2 y]	
Diebold et al. (2019)^8^	F	68	Ps	6	UVA, UVB, MTX, FAE	SEC	RRMS	No (exacerbation)	Prednisolone	PASI100 (2 mo); paraparesis, new T2 MRI lesions (10 mo)	SEC was replaced from rituximab after MS relapse
Di Tulio et al. (2020)^13^	M	44	Ps, PsA	11	ETA, INF, ADA, UST, CER, GOL	SEC 300 mg (at wk 0, 1, 2, 3, 4, then monthly) /150 mg after MS onset	RRMS	Yes, 2 y after treatment initiation	DMF (240 mg BID), after MS onset	PASI100 and articular involvement improved (3 mo); plantar hypoesthesia, paraesthesia of the upper limbs, and MRI focal demyelinated areas (2 y)	Up to 12 mo after administration of both treatments, good control of all diseases was observed
Herrera‐Acosta et al. (2021)^9^	M	50	Ps	40	MTX, acitretin, UST	IXE 80 mg	MS	No		From PASI 14 to 3 (4 wks) and to 0; MS stable [follow‐up 3 y]	
Kougkas et al. (2022)^10^	F	Early 30s	Ps, PsA	5	ADA, MTX	SEC 300 mg monthly	MS	No	Prednisolone, glatiramer acetate, DMF	Ps, PsA: low disease activity (16 mo); MS relapse (19 mo)	After relapse: natalizumab and SEC, clinical improvement for all conditions
Megna et al. (2020)^11^	M	47	Ps, PsA	25	MTX, cyclosporine	SEC 300 mg (weekly for the first 5 wks, every 4 wks thereafter)	MS	No	No	PASI90 (6wks); MS: stable [follow‐up 48 wks]	
Romozzi et al. (2021)^14^	M	28	Ps, PsA	18	ADA	IXE	Myelitis	Yes, 10 mo after 1st treatment initiation, 6 mo after 2nd treatment initiation	No	Subacute mild right leg hyposthenia (1st:10 mo, 2nd: 6 mo)	
Venturini et al. (2020)^12^	F	45	Ps, PsA	16	Cyclosporine A, narrowband UVB, ETA, ADA, GOL, UST, INF, APR	SEC 300 mg (once every 4 wks)	MS	No	No	PASI90 (6 wks); PASI100 (12 wks); MS: stable [follow‐up 2 y]	

Abbreviations: ADA, adalimumab; APR, apremilast; BID, twice daily; CER, certolizumab pegol; DMF, dimethyl fumarate; ETA, etanercept; F, female; FAE, fumaric acid esters; GOL, golimumab; INF, infliximab; IXE, ixekizumab; M, male; mo, months; MS, multiple sclerosis; MTX, methotrexate; PASI, Psoriasis Area Severity Index; Ps, psoriasis; PsA, psoriatic arthritis; RRMS, relapsing remitting multiple sclerosis; SEC, secukinumab; UST, ustekinumab; UVA, Ultraviolet (UV) light, UVA rays; UVB, Ultraviolet (UV) light, UVB rays.

* until treatment initiation.

^a^ Outcomes under anti‐IL‐17 biologic treatment.

The use of anti‐IL‐17 biologics for psoriasis and concomitant pre‐existing MS has been reported in six case reports.^7^, ^8^, ^9^, ^10^, ^11^, ^12^ Five of them^7^, ^9^, ^10^, ^11^, ^12^ reported anti‐IL‐17‐mediated satisfactory control of both the neurological and skin symptoms. In three cases^9^, ^11^, ^12^ biologics were administered as a monotherapy, while in two cases^7^, ^10^ patients were treated with anti‐IL‐17 biologics combined with other agents (prednisolone, methotrexate, glatiramer acetate, dimethyl fumarate, and natalizumab).

However, in the sixth report^8^ MS was not adequately controlled. Specifically, a 68‐year‐old female, who had been experiencing psoriatic skin lesions for 6 years and had been exhibiting episodes of motor or visual symptoms for at least 4 years was prescribed secukinumab. While treatment induced a skin response, the patient experienced a neurological relapse. Whether secukinumab mediated an exacerbation of the pre‐existing neurological condition, or did not manage to effectively attenuate its manifestations, could not be concluded.

Another report presented a 44‐year‐old male who had been diagnosed with psoriasis 11 years ago and had received various treatments without achieving an adequate response.^13^ The patient responded to secukinumab, but 2 years later was diagnosed with RRMS. The patient received DMF and secukinumab and maintained a good clinical response up to 12 months later.

A case of a 28‐year‐old male presenting myelitis after exposure to ixekizumab has also been reported.^14^ The patient had been unsuccessfully treated with adalimumab and was later switched to ixekizumab. However, 10 months after initiation mild hyposthenia of the right leg developed. Treatment discontinuation and administration of prednisolone resulted in clinical improvement. Two years later, the patient was reintroduced to ixekizumab because of relapsing skin lesions. Hyposthenia of the lower limb was re‐experienced. A spinal MRI showed a hyperintense lesion in T2 while various non‐specific lesions were detected on brain MRI.

## 4 CONCLUSION

Our review showed that, in most cases, secukinumab or ixekizumab administration controlled skin and neurological manifestations.

No association between anti‐IL‐17 biologics and demyelination has been established. On the contrary, inhibition of IL‐17 has been considered an important therapeutic target in patients with psoriasis and concomitant MS since the pathophysiology of psoriasis implicates mechanisms perturbing the IL‐23/Th17 axis. Specifically, in psoriatic skin activated dendritic cells produce TNF‐α, a landmark mediator of the disease and an upstream cytokine of the axis, which potentiates a pro‐inflammatory milieu that induces IL‐23 expression. TNF is also associated with skin‐homing chemokines that maintain IL‐17‐producing cells in the epidermis. Produced IL‐23 facilitates the maintenance and expansion of Th17 cells. Interestingly, agents that have been used for MS, such as DMF, have also been considered effective for psoriasis.

Our patient had been vaccinated against SARS‐CoV‐2 3 months prior to the occurrence of neurological manifestations. There have been cases presenting similar symptoms to what our patient experienced after vaccination.^15^ Whether vaccination in isolation or in combination with ixekizumab treatment induced de novo or accelerated an underlying myelin‐specific autoreactive response leading to MS remains unknown. An epiphenomenal association, either with ixekizumab or SARS‐CoV‐2 vaccination, cannot be ruled out, given that the patient developed MS, 8 months after the initiation of ixekizumab and 3 months after the second dose of the mRNA SARS‐CoV‐2 vaccine.

Moreover, there have been reports suggesting a rare association between psoriasis and MS, or transverse myelitis and psoriatic arthritis.^16^ Thus, we can not exclude the probability of coincidental onset of MS during the course of psoriasis independently from treatment received.

While the anti‐IL‐17‐biologic‐mediated induction of inflammatory events in CNS has not been proven and a causal relationship is lacking, such a probability should not be ruled out in genetically prone individuals. Recognition of the clinical signs by dermatologists and prompt evaluation could lead to proper treatment and improved clinical outcomes for patients.

## CONFLICT OF INTEREST STATEMENT

Dimitrios P. Bogdanos declares lecture honoraria (2020–2023) from Abbvie Pharmaceuticals SA, Novartis, Menarini Hellas, Boehringer Ingelheim, Genesis Pharma, Pharmaserv‐Lilly, Fresenius Kabi, Euroimmun, Werfen, Sobi; congress travel and accommodation support from Abbvie, Novartis, Hospital Line, Pfizer, Elpen, Aenorasis Hellas, Pharmaserv‐Lilly, Sobi. Efterpi Zafiriou declares consulting fees from Abbvie, Genesis Pharma, Leo, Novartis, Jannsen, Pharmaserv Lilly, Pfizer, Sanofi, UCB, payment or honoraria: Abbvie, Genesis Pharma, Leo, Novartis, Jannsen, Pharmaserv Lilly, Pfizer, Sanofi, UCB, support for attending meetings: Abbvie, Genesis Pharma, Novartis, Jannsen, Pharmaserv Lilly, Sanofi, UCB. Sotirios G. Tsiogkas, Vaia Tsimourtou, Kleoniki Chaidaki, Efthymios Dardiotis, and Angeliki Victoria Roussaki‐Schulze declare no conflicts of interest.

## ETHICS STATEMENT

Written informed consent was obtained from the patient for publication of this report.
